# Celiac Artery Compression Syndrome: An Experience in a Single Institution in Taiwan

**DOI:** 10.1155/2012/935721

**Published:** 2012-09-04

**Authors:** Jen-Wei Chou, Chih-Ming Lin, Chun-Lung Feng, Chun-Fu Ting, Ken-Sheng Cheng, Yung-Fang Chen

**Affiliations:** ^1^School of Medicine, China Medical University, Taichung 40402, Taiwan; ^2^Division of Gastroenterology and Hepatology, Department of Internal Medicine, China Medical University Hospital, 2 Yude Road, North District, Taichung 40447, Taiwan; ^3^Department of Radiology, China Medical University Hospital, Taichung 40447, Taiwan

## Abstract

Celiac artery compression syndrome (CACS) or median arcuate ligament (MAL) syndrome is a rare vascular disease. The clinical manifestations of CACS include the triad of postprandial pain, vomiting, and weight loss. The pathogenesis of CACS is the external compression of celiac artery by the MAL or celiac ganglion. Moreover, some authors also reported the compression with different etiologies, such as neoplasms of pancreatic head, adjacent duodenal carcinoma, vascular aneurysms, aortic dissection, or sarcoidosis. In the literature, most cases of CACS were reported from Western countries. In contrast, this disease was seldom reported in Oriental countries or regions, including Taiwan. Superior mesenteric artery syndrome (SMAS) is also a rare disease characterized by compression of the third portion of the duodenum by the SMA. The clinical features of SMAS are postprandial pain, vomiting, and weight loss. To date, there are no guidelines to ensure the proper treatment of patients with CACS because of its low incidence. Thus, tailored therapy for patients with CACS remains a challenge as well as the prediction of clinical response and prognosis. The aim of our present study was to investigate the clinical features, the association with SMAS, treatments, and outcomes of patients with CACS in a single institution in Taiwan.

## 1. Introduction

Celiac artery compression syndrome (CACS), also called median arcuate ligament (MAL) syndrome or Dunbar syndrome, is a rare vascular disease [1–3]. As early as the 1960s, Dunbar first described the stenotic anomaly of celiac trunk through visualization angiographically. He correlated the image findings and clinical manifestations of patients presenting with abdominal angina, such as postprandial pain, nausea/vomiting, and weight loss. He found that those patients became symptom-free after successful surgical release of the compression by sectioning the MAL. Although the existence of CACS remains controversial, it is widely accepted that CACS is a disease of mesenteric ischemia and should be differentiated with other ischemic bowel diseases. The pathogenesis of CACS is the external compression of celiac artery by the MAL or celiac ganglion [4]. It has caught the attention of many authors who are attempting to figure out the relationship of celiac artery and diaphragm in embryogenesis and hereditary [5]. Moreover, some authors also reported the compression with different etiologies, such as neoplasms of pancreatic head, adjacent duodenal carcinoma, vascular aneurysms, aortic dissection, or sarcoidosis [6]. In the literature, most cases of CACS were reported from Western countries. In contrast, this disease was seldom reported in Oriental countries or regions. In Taiwan, we reported two cases of CACS previously [7, 8]. Superior mesenteric artery syndrome (SMAS), first described in 1842 by Carl Freiherr von Rokitansky, is also a rare disease characterized by compression of the third portion of the duodenum by the SMA [9]. For normal patients, the aortomesenteric angle is 28 to 65 degrees and the aortomesenteric distance is from 10 to 34 mm [10]. For patients with SMAS, the aortomesenteric angle is less than 25 degrees (6°–25°) or the aortomesenteric distance is less than 8 mm. The clinical features of SMAS are postprandial pain, vomiting, and weight loss [9].

To date, there are no guidelines to ensure the proper treatment of patients with CACS because of its low incidence. Thus, tailored therapy for patients with CACS remains a challenge as well as the prediction of clinical response and prognosis. The aim of our present study was to investigate the clinical features, the association with SMAS, treatments, and outcomes of patients with CACS in a single institution in Taiwan. Additionally, we also reviewed the related literature of this rare disease. 

## 2. Patients and Methods

**Figure 1:**
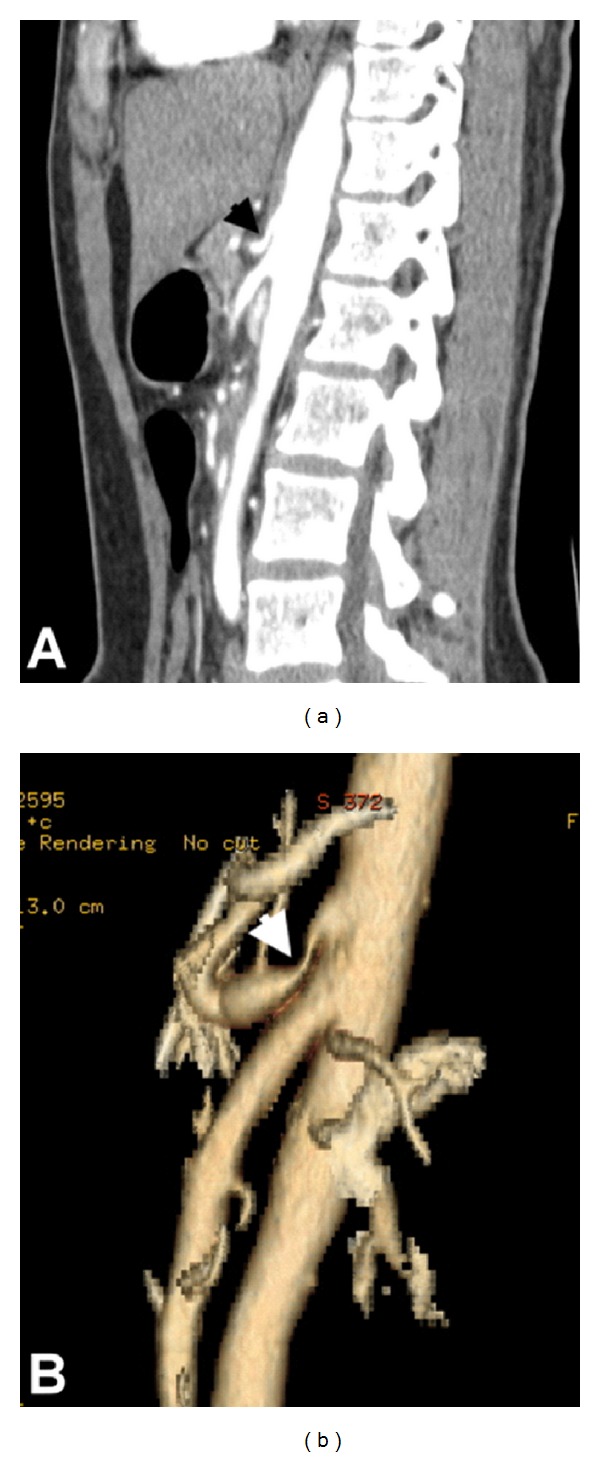
Computed tomography angiography of the celiac artery compression syndrome demonstrated the characteristic hooked narrowing of the proximal celiac artery with poststenotic dilatation in 2D reconstruction image (a, arrow) and 3D reconstruction image (b, arrow).

**Figure 2:**
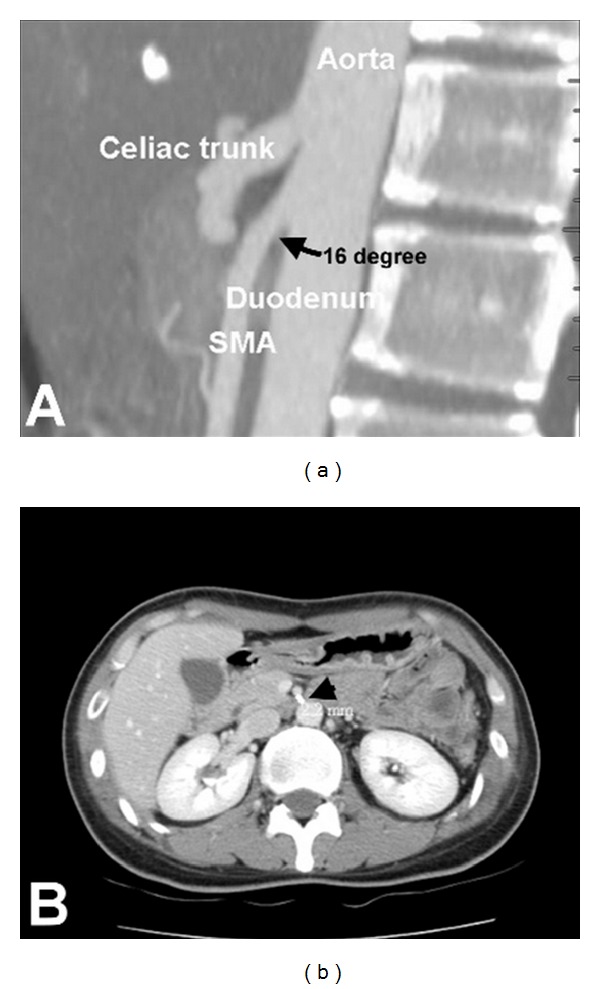
A diagnostic criteria of superior mesenteric artery syndrome are based on the aortomesenteric angle less than 25° (a, arrow) or aortomesenteric distance less than 8 mm (b, arrow).

From January 2003 to March 2011, we retrospectively reviewed the medical records of patients who were diagnosed as CACS in the outpatient department and inpatient department of China Medical University Hospital, a tertiary referral hospital in the middle of Taiwan. The diagnostic criteria of CACS were based on patients with the chief complaints of postprandial pain, nausea/vomiting, or weight loss in addition to the typical findings of computed tomography angiography (CTA). The multidetect 16-slice abdominal CT scanner with postprocedure 2D and/or 3D reconstruction was performed. The typical findings of CACS in CTA include hook appearance in the proximal portion of celiac artery, with a poststenotic dilatation in the distal portion (Figures 1(a) and 2(b)). Moreover, the angle between the aorta and the SMA was calculated. The aortomesenteric angle less than 25° or the aortomesenteric distance less than 8 mm with the typical symptoms was considered to a definite diagnosis of SMAS (Figures 2(a) and 2(b)). Age, gender, clinical symptoms, body mass index (BMI), comorbid diseases, diagnostic modalities, treatments, and outcomes of patients with CACS were also analyzed. Moreover, we also discussed the association of CACS and SMAS.

In the statistical analysis, data were expressed as mean (standard error of mean) with range, when appropriate.

## 3. Results

A total of 14 patients diagnosed as the CACS were enrolled into our current study (see Tables 1 and 2). With regard to age and gender: the mean age of total 14 patients was 28.4 years ± 10.9, ranging from 18 to 53 years. In a subgroup analysis, 10 were female (71%), ranging from 18 to 51 years, with a mean of 26.4 years; 4 were male (29%), ranging from 23 to 53 years, with a mean of 33.5 years. With regard to BMI: it ranged from 14.3 to 21.2 kg/m^2^, with a mean of 18.2 ± 1.9 kg/m^2^. The mean BMI of female patients was 18.3 kg/m^2^, while the mean BMI of male patients was 18.1 kg/m^2^. As to clinical symptoms: all patients (100%) presented with postprandial pain, 8 patients (57%) presented with nausea/vomiting, and 5 patients (36%) presented with body weight loss. For comorbidities: almost all of the patients had no chronic medical diseases except for one patient that had juvenile rheumatoid arthritis. Furthermore, the coexistence of CACS and SMAS was identified in 9 out of 14 patients (64%). As to treatment, two patients with CACS and SMAS underwent surgical decompression treatment. One underwent laparotomy with division of MAL; the other one underwent laparoscopic division of MAL initially, but it was converted to laparotomy due to iatrogenic bleeding of the celiac artery. The former had a good outcome on the postoperative followup; the latter had recurrent symptoms 3 months later after operation. In addition, one patient underwent percutaneous transluminal angioplasty (PTA), but this failed to relieve symptoms. In the conservative treatment group, 11 patients had the recurrent symptoms at a median followup of 4 years.

## 4. Discussion

Although CACS has been introduced to people for several decades, it is not well understood until recently. In Western populations, the incidence of CACS was reported to ranging from 12.5–24% [6, 11–13]. However, a very low incidence rate of 2.3% has been reported in Japan [14]. The disease usually occurs in young patients with a female predominance [2, 15]. In comparison to the previous reports from Western countries, our patients were younger with a mean age of 28.4 ± 10.9 years. Moreover, the female accounted for 71% of all patients and was younger than male patients in our study. CACS and SMAS usually occur in patients with a low BMI. In the current study, our patients had a low mean BMI of 18.2 ± 1.9 kg/m^2^ with an equal distribution in both sexes. 

The clinical manifestations of CACS include the triad of postprandial pain, nausea/vomiting, and weight loss [2]. In our present study, the postprandial pain is the most common symptom in all patients. However, these symptoms are usually nonspecific and are easily misdiagnosed as functional dyspepsia, peptic ulcer disease, or gastropathy. Patients will not present with symptoms if compensated well via the collateral vessels or the blood flow is sufficient to handle demands. There are two main theories used to explain the pathogenesis of the symptoms. The first theory is mesenteric ischemia arises either from direct foregut ischemia or, alternatively, through postprandial steal via collaterals from the superior mesenteric artery to the celiac bed, leading to midgut ischemia [16]. The second theory is neurogenic stimulation caused by direct compression of the celiac ganglion and plexus, leading to splanchnic vasoconstriction or via direct sympathetic pain fiber irritation [17].

Although CACS and SMAS are two rare different disorders with an uncertainty of pathogenesis, they share similar clinical symptoms. However, their association was seldom studied in the literature. Sianesi et al. reported 59 patients affected by CACS and 28 by SMAS [18]. The coexistence of both syndromes in 8 patients was observed in their study. In our present study, we identified the coexistence of CACS and SMAS in 64% of all patients. Based on our experiences in the current study, we hypothesize that CACS may be a rare etiology of SMAS because of the mesenteric ischemia of CACS can induce the weight loss of patients and result in the formation of SMAS. 

The diagnosis of CACS is usually based on typical clinical symptoms with radiological imaging. Lateral view aortography had been thought to be a golden standard modality in diagnosing CACS [19]. However, it is invasive, expensive, and time consuming. Moreover, CACS may be misdiagnosed if only an anterior-posterior view is obtained. Scholbach reported that color Doppler ultrasound might be a powerful tool for CACS screening [20]. However, its role in diagnosing CACS is still controversial because of the influence of iatrogenic factors and the high degree of dependence on the technicians' ability and experience. Recently, multidetector CTA with proper 2D or 3D postprocessing techniques has become a more favorable modality in diagnosing vascular diseases, including CACS and SMAS [21, 22]. CTA can demonstrate the presence and degree of stenosis of the celiac artery and SMA, the collateral circulation, relationships between vessels and adjacent tissues, and excluding other causes of vascular obstruction. The classical findings of CACS in CTA include thickened MAL, asymmetrical, and respiratory-dependent stenosis of proximal celiac artery with poststenotic dilatation, hooked appearance of celiac artery with indentation of adjacent aorta [21]. In addition to the radiological imaging, a functional test ideally should be present to prove the presence of mesenteric ischemia. Currently, only PC2 tonometry or visible light spectroscopy has been validated [23]. 

In the treatment of CACS, there were a number of surgical approaches and endovascular therapies outlined in the literature. Conventional open surgery (either transabdominal by median laparotomy or retroperitoneal by left subcostal incision), including division of the MAL and/or resection of periarterial neurofibrotic tissue, is usually adequate in most patients [2, 24]. Moreover, some authors suggested additional arterial reconstruction of the entrapped celiac artery by primary reanastomosis, interposition grafting, or bypass to offer better outcome [25, 26]. The average rate of being symptom-free is around 70–80% after successful surgery based on long-term followup [26]. Although the success rates of open surgery on vascular patency are excellent, the operative trauma to the abdominal wall and cavity is extensive. Recently, laparoscopy is considered a novel approach for the treatment of CACS. In 2000, Roayaie et al. performed the first laparoscopic release of CACS [27]. In 2009, Baccari et al. reported a case series study, in which 14 of 16 patients remained symptom-free in the followup after laparoscopic approach [28]. Moreover, van Petersen et al. first applied an endoscopic retroperitoneal approach instead of an abdominal endoscopic approach for the release of the celiac trunk in CACS [29]. The reason was that retroperitoneal method can visualize the complex local anatomy more distinctly. The laparoscopic approach has the advantage of being less invasive but equally effective for decompressing the entrapped celiac artery. Moreover, it avoids the morbidity of an upper-midline laparotomy and shortens hospital stay, resulting in early refeeding [30]. Although there are more patients with CACS receiving the laparoscopic approach, there is potential risk of vascular injury through using the method such as our patient 12, and adjunctive celiac artery intervention is often required. Therefore, surgical approaches, both laparoscopic and open, can be safely performed with minimal morbidity and mortality. Endovascular therapies for revascularization of celiac artery by PTA with either balloon dilatation or stent implantation have been reported in the literature [31–33]. Furrer et al. first reported PTA in treating mesenteric ischemia in 1980 [31]. In reports thereafter, almost all patients were free of symptoms immediately after the intervention. However, the recurrence rate was high, and the duration of being symptom-free was relatively short. Hence, PTA and stenting in treating CACS are usually unsuccessful because the extrinsic pressure on the celiac artery (the surrounding fibrotic tissue or MAL) will result in the slippage of the stent and/or damage to their material. Prior surgical decompression followed by consolidation of stent implantation is optimal [33]. Therefore, endovascular therapy could play a role of adjuvant therapy or bridging to surgery in treating CACS. In our study, surgical and endovascular treatments were seldom performed in patients with CACS. Only three patients underwent invasive treatment in our present study. The main reason is not enough of an understanding of this disease for most surgeons and gastroenterologists. Patient 1 underwent PTA therapy but had poor response. Patient 2 underwent open laparotomy and had a favorable outcome. CTA demonstrated no stenosis of celiac artery in the postoperative followup. Patient 12 underwent laparoscopic resection of periarterial fibrotic tissue initially; however, it was converted to open surgery due to vascular injury. She had recurrent symptoms 3 months later after operation. In contrast, conservative medical treatment usually has no satisfactory benefits for CACS as in our study.

In the prognosis, a large study reported that 83% of patients with CACS were asymptomatic in the first 6 months after decompression, but only 41% of patients remained asymptomatic 3 to 11 years later [34]. Although late recurrence is frequently seen, this seems to be milder than the presenting symptoms. In another study reported by Reilly et al., patients with CACS were symptom-free with a mean of 9 years after surgery [25]. Because the number of surgical treatment is too small in our study, we need more available long-term follow-up data for patients with CACS after surgery.

## 5. Conclusions

CACS is a rare vascular disease clinically, it should be considered in the group of young female patients with abdominal symptoms and a low BMI in Taiwan. CTA is a powerful modality in diagnosing this disease. Although surgical intervention is a mainstay choice in treating patients with CACS, it is seldom performed in Taiwan because most clinicians lack experiences of this disease. To date, this present study is a large case series of CACS in Oriental countries.

## Figures and Tables

**Table 1:** The demographic and clinical characteristics of patients with celiac artery compression syndrome (*n* = 14).

Case number	Gender	Age (y/o) mean: 28.4 ± 10.9	BMI (kg/m^2^) mean: 18.2 ± 1.9	Symptoms
1	Male	34	18.3	Postprandial epigastric pain, weight loss
2	Female	27	14.3	Postprandial epigastric pain, nausea/vomiting, weight loss
3	Female	23	17.4	Postprandial epigastric pain, weight loss
4	Female	19	15.4	Postprandial epigastric pain, nausea/vomiting
5	Male	23	19.9	Postprandial epigastric pain, nausea/vomiting
6	Female	25	18.4	Postprandial epigastric pain, nausea/vomiting, weight loss
7	Male	24	16.8	Postprandial epigastric pain, nausea/vomiting
8	Female	51	19.5	Postprandial epigastric pain
9	Male	53	17.4	Postprandial epigastric pain, weight loss
10	Female	25	19.8	Postprandial epigastric pain
11	Female	22	17.4	Postprandial epigastric pain, nausea/vomiting
12	Female	32	19.7	Postprandial epigastric pain, nausea/vomiting
13	Female	18	21.2	Postprandial epigastric pain, nausea/vomiting
14	Female	22	19.9	Postprandial epigastric pain

BMI: body mass index.

**Table 2:** The diagnosis modalities, treatments, and outcomes of patients with celiac artery compression syndrome (*n* = 14).

Case number	Diagnostic modality	Coexist SMAS	Treatment	Outcome
1	CTA	No	PTA	Recurrence
2	CTA	Yes	Laparotomy	Cure
3	CTA	Yes	Conservative	Recurrence
4	CTA	No	Conservative	Recurrence
5	CTA	Yes	Conservative	Recurrence
6	CTA	No	Conservative	Recurrence
7	CTA	Yes	Conservative	Recurrence
8	CTA	Yes	Conservative	Recurrence
9	CTA	No	Conservative	Recurrence
10	CTA	No	Conservative	Recurrence
11	CTA	Yes	Conservative	Recurrence
12	CTA	Yes	Laparoscopy + laparotomy	Recurrence
13	CTA	Yes	Conservative	Recurrence
14	CTA	Yes	Conservative	Recurrence

CTA: computed tomography angiography; PTA: percutaneous transcatheter angioplasty; SMAS: superior mesenteric artery syndrome.
